# Low PCSK9 levels are correlated with mortality in patients with end-stage liver disease

**DOI:** 10.1371/journal.pone.0181540

**Published:** 2017-07-20

**Authors:** Valentin Schlegel, Theresa Treuner-Kaueroff, Daniel Seehofer, Thomas Berg, Susen Becker, Uta Ceglarek, Joachim Thiery, Thorsten Kaiser

**Affiliations:** 1 Institute of Laboratory Medicine, Clinical Chemistry and Molecular Diagnostics, University Hospital Leipzig, Leipzig, Germany; 2 Department of Visceral, Vascular, Thoracic, and Transplant Surgery, University Hospital Leipzig, Leipzig, Germany; 3 Section of Hepatology, Department of Gastroenterology and Rheumatology, University Hospital Leipzig, Leipzig, Germany; Centro Cardiologico Monzino, ITALY

## Abstract

**Introduction:**

Proprotein convertase subtilisin/kexin type 9 (PCSK9) plays a key role in the cholesterol metabolism and is synthesized by the liver. It interacts with the LDL-receptor to promote its degradation. The model of end-stage liver disease (MELD) score is a well-established tool to estimate the risk of mortality in patients with end-stage chronic liver disease. The study aims to assess the associations between PCSK9, hypocholesterinemia, liver synthesis, cholestasis, MELD score and mortality in patients with end-stage liver disease.

**Methods:**

Serum samples were obtained from 74 patients with severe liver disease. The study participants were aged between 23 and 70 y (mean: 55.8 y; 47 males [63.5%], 27 females [36.5%]). Samples were selected from those with a wide range of MELD scores (7 to 40).

Patients that underwent liver transplantation (17 / 74) were censored at the time of transplantation for mortality analysis.

**Results:**

PCSK9 values ranged from 31.47 ng/mL to 261.64 ng/mL. The median value was 106.39 ng/ml. PCSK9 was negatively correlated with markers of liver function and cholestasis (INR, bilirubin). Over a 90-d follow-up, 15 of 57 (26,3%) patients died within the 90-d follow-up without receiving liver transplantation. Thirteen of 31 (42%) patients with PCSK9 levels below the median died compared to 2/26 (8%) patients with higher PCSK9 levels (*p* = 0.006). In this cohort, there were no significant correlations between PCSK9, cholesterol, its precursors and several phytosterols.

**Conclusions:**

Low PCSK9 serum concentrations were associated with higher mortality in patients with end-stage liver disease. The mean PCSK9 levels in the study population were much lower than those found in normal or healthy populations. Further studies are required to acquire a more detailed understanding of the role of PCSK9 in liver-related mortality.

## Introduction

Proprotein convertase subtilisin/kexin type 9 (PCSK9) is a member of the proprotein convertase family and encodes a protein with 692 amino acids [1,2]. It is mainly synthesized in the liver but can also be found in kidneys and intestines [3]. Abifadel et al. discovered that a mutation in the PCSK9 gene was the cause of a form of autosomal dominant hypercholesterolemia [1]. Several research teams showed that different gain-of-function mutations in PCSK9 led to higher levels of plasma low-density lipoprotein (LDL) cholesterol and were associated with coronary heart disease [2,4–6]. PSCK9 is synthesized by the liver and secreted in the blood [7]. In a study of liver-specific PCSK9 knockout mice, Cariou et al. did not detect any PCSK9 circulating in the blood, suggesting that liver-synthesized PCSK9 was the only source of PCSK9 in the blood [8]. The mechanisms involved in the expression of PCSK9 are not fully understood. However, sterol regulatory element binding protein 2 (SREBP2) seems to play an important role, as it promotes the expression of PCSK9. The activation of SREBPs is linked to intracellular sterol levels, with activation occurring in response to decreases in these levels [9]. Interestingly, SREBP2 promotes the expression of both LDL receptor (LDLR) and PCSK9, which plays a key role in the regulation of the LDLR [10]. PCSK9 binds to the LDLR on the cell surface and after internalization of the receptor, it inhibits endocytic recycling of the LDLR, resulting in the degradation of both LDLR and PCSK9 in lysosomes [11–14]. Therefore, PCSK9 has a major influence on cholesterol homeostasis in the body, and it has become an important target for new drugs to lower blood cholesterol [15].

In recent years, anti-PCSK9 monoclonal antibodies were developed that blocked the interaction between PCSK9 and the LDLR at the cell surface [16,17]. These new drugs led to considerable lowering of plasma LDL cholesterol concentrations [15,18]. However, low cholesterol levels have been linked to reduced liver regeneration capacity and increased mortality in patients with end-stage liver disease [19]. To date, little has been published on the synthesis and function of PCSK9 in the context of liver cirrhosis and prognosis.

The model of end-stage liver disease (MELD) score is a well-established tool to estimate the risk of mortality in patients with end-stage chronic liver disease [20]. It was first developed as a model to predict poor survival in patients scheduled to receive a transjugular intrahepatic portosystemic shunt and is used to prioritize patients requiring a liver transplant [21]. The MELD score is based on serum bilirubin levels, serum creatinine levels, and the international normalized ratio (INR) and is used as a liver disease severity index [22]. It has been validated in diverse liver disease patient populations, including those with a wide range of etiologies, as well as in patients with different stages of disease severity [21,22].

Hypocholesterolemia is known to be associated with a worse prognosis in patients with end-stage liver disease. Thus, the aim of the present study was to assess the associations between PCSK9, liver synthesis, cholestasis, and mortality.

## Methods

Serum samples were obtained from 74 patients with severe liver disease. The study participants were aged between 23 and 70 y (mean age: 55.8 y; 47 males [63.5%], 27 females [36.5%]). To include patients with different stages of chronic liver disease, samples were selected from those with a range of MELD scores. The MELD scores of the included patients ranged from 7 to 40, and the mean score was 21. There were different underlying etiologies of liver disease: 46 patients (62.2%) had alcohol-induced liver disease, 12 (16.2%) nonalcoholic steatohepatitis, 2 (2.7%) hepatitis C virus infection, 2 (2.7%) Budd-Chiari syndrome, 2 (2.7%) autoimmune hepatitis, 1 (1.4%) primary sclerosing cholangitis, 1 (1.4%) drug-induced, 1 (1.4%) hemochromatosis, 1 (1.4%) cholangiocellular carcinoma, 1 (1.4%) hemosiderosis, 6.5% cryptogenic cirrhosis. We did not include patients with acute liver failure. The patients were followed up for 90 d after blood taking. For mortality analysis, patients who received a transplant were censored at the time of the transplantation. The ethics committee of the University Hospital Leipzig approved the utilization of residual citrated plasma samples for this study without an additional informed consent (ethical approval 082-10-190-42010).

A commercial ELISA (enzyme-linked immuno-sorbent assay) assay kit (Quantikine ELISA, R&D Systems, Mineapolis, MN, USA) was used for the measurement of PCSK9. Serum levels of analytes were measured according to the manufacturer's specifications using a Cobas 8000 Analyzer (Roche, Mannheim, Germany): bilirubin (Total DPD Gen.2 kit, Roche, Mannheim, Germany), sodium (ISE indirect NA-K-Cl for Gen.2, Roche, Mannheim, Germany), creatinine (Creatinine Plus Ver.2 kit, Roche, Mannheim, Germany), gamma-glutamyl transferase (GGT-2/γ-Glutamyltransferase ver.2, Roche, Mannheim, Germany), aspartate transaminase (AST) (ASTPM, Aspartate Aminotransferase, Roche, Mannheim, Germany), alanine aminotransferase (ALT) (ALTPM, Roche, Mannheim, Germany), alkaline phosphatase (AP) (ALP2, Roche, Mannheim, Germany) and cholinesterase (CHE) (CHE2, Roche, Mannheim, Germany). The INR was determined in citrate plasma using the ACL TOP 700 System (Instrumentation Laboratory, Lexington, Massachusetts, USA).

Sterols were determined based on the previously published liquid chromatography–mass spectrometry (LC-MS/MS) method [23]. In brief, 10 μl of calibrator, quality control, or sample (serum or plasma) were mixed with 490 μl of internal standard solution (100 μg/L d7-cholesterol in methanol/isopropanol, 1/1, v/v) in polypropylene tubes. Following centrifugation for 10 min at 11,400 g, the supernatant was transferred to glass vials for tandem mass spectrometric analysis. Then, 25 μl of the supernatants were injected into an analytical 50 × 4.6 mm, monolithic column (Chromolith SpeedROD RP-18e, Merck KGaA, Darmstadt, Germany). An API 4000 triple quadrupole mass spectrometer with an atmospheric pressure photoionization source (AB Sciex, Darmstadt, Germany) with an atmospheric pressure photoionization source was used.

### Statistical analysis

Data were analyzed using Excel 14.0 (Microsoft Corporation, Redmond, Washington, U.S.A) and SPSS 23.0 (IBM, Armonk, New York, U.S.A). Spearman’s rank correlation coefficient was calculated. Mortality analysis was performed by logistic regression analysis and Kaplan–Meier curves. A two-sided chi square test was used to investigate whether distributions of categorical variables differed from each other. In the statistical analysis, a p value <0.05 was considered statistically significant.

## Results

PCSK9 values ranged from 31.47 ng/mL to 261.64 ng/mL, and the mean value was 111.57 ng/mL. Analysis of the correlation of PCSK9 with liver synthesis parameters, cholestasis, and other mortality-related parameters (i.e., MELD scores) was performed and revealed significant correlations between PCSK9 and the markers of liver function (Table 1). The examination showed positive correlations between PCSK9 levels and albumin and cholinesterase. Correspondingly, PCSK9 and INR were negatively correlated with each other.

The parameters of cholestasis and hepatic detoxification (total bilirubin, conjugated bilirubin, and unconjugated bilirubin) were negatively correlated with PCSK9 serum concentrations. In contrast, γ-glutamyl transferase showed a significant correlation with PCSK9 serum concentrations. The correlation of alkaline phosphatase with PCSK9 serum concentrations was not significant.

**Table 1:** Correlation coefficients for PCSK9 with biomarkers of cholesterol synthesis and liver function. (The correlation was significant at the *p = 0.05, **p = 0.01 and ***p = 0001 level).

	Correlation Coefficient	N
**PCSK9 (ng/mL)**	1	74
**MELD Score**	-0.438^***^	74
**International Normalized Ratio (INR)**	-0.577^***^	74
**Albumin (g/L)**	0.243^*^	74
**Cholinesterase (μkat/L)**	0.409^***^	74
**Bilirubin total (μmol/L)**	-0.416^***^	74
**Bilirubin conjugated (μmol/L)**	-0.396^***^	70
**Bilirubin unconjugated (μmol/L)**	-0.595^***^	62
**Creatinine (μmol/L)**	0.054	74
**Alanine Transaminase (ALT) (μkat/L)**	0.051	74
**Aspartate Transaminase (AST) (μkat/L)**	-0.168	73
**γ-Glutamyl Transferase (μkat/L)**	0.425^***^	74
**Alkaline Phospatase (μkat/L)**	0.119	74
**Brassicasterol (mg/L)**	-0.016	74
**Campesterol (mg/L)**	-0.037	74
**Stigmasterol (mg/L)**	-0.154	74
**Sitosterol (mg/L)**	-0.148	74
**Lanosterol (mg/L)**	0.122	74
**Cholesterol (mg/L)**	0.119	74
**Desmosterol+Zymosterol+7-Dehydrocholesterol (mg/L)**	-0.094	74

Markers of liver damage, such as ALT and AST, as well as creatinine, a marker of renal function, were not significantly correlated with PCSK9 levels.

We further investigated the correlation between PCSK9 concentrations and those of cholesterol precursors (lanosterol and the sum of desmosterol, zymosterol, and 7-dehydrocholesterol). The concentrations of these precursors (lanosterol, desmosterol, zymosterol, and 7-dehydrocholesterol) were not significantly associated with PCSK9 concentrations. Furthermore, analysis of the association between PSCK9 and phytosterols (brassicasterol, campesterol, stigmasterol, and sitosterol) revealed no significant correlation. Table 2 shows the results of the biomarkers for patients with PCSK9 values above and below the median value.

**Table 2:** Comparison of the levels of different biomarkers in patients with PCSK9 levels above and below the median value.

	PCSK9 < Median			PCSK9 > Median			
	Median	Maximum	Minimum	Median	Maximum	Minimum	p
**PCSK9 (ng/mL)**	77.18	104.46	31.47	140.44	261.64	108.32	< 0.001
**MELD Score**	25.38	48.92	10.46	15.13	39.27	6.53	0.001
**International Normalized Ratio (INR)**	1.99	4.84	1.25	1.28	3.51	1	< 0.001
**Albumin (g/L)**	31.50	46.50	19.60	36	47.8	19	0.054
**Cholinesterase (μkat/L)**	25.20	100.80	5.50	44.3	146.7	9.5	0.010
**Bilirubin total (μmol/L)**	119.60	640.60	6.10	20.7	724.9	5.3	< 0.001
**Bilirubin conjugated (μmol/L)**	65.30	427.40	11.60	9.7	486.8	3	< 0.001
**Bilirubin unconjugated (μmol/L)**	42.60	160.20	6.20	6.8	158.1	1.1	< 0.001
**Creatinine (μmol/L)**	104.00	398.00	39.00	108	390	37	0.336
**Alanine Transaminase (ALT) (μkat/L)**	0.34	58.09	0.11	0.32	3.3	0.1	0.779
**Aspartate Transaminase (AST) (μkat/L)**	1.07	10,00	0.47	0.76	10.35	0.38	0.106
**γ-Glutamyl Transferase (μkat/L)**	0.82	7.38	0.23	1.75	18.85	0.24	0.001
**Alkaline Phospatase (μkat/L)**	1.91	6.59	0.66	2.1	6.84	0.9	0.452
**Brassicasterol (mg/L)**	2.70	6.41	0.49	2.19	5.5	0.24	0.341
**Campesterol (mg/L)**	9.85	22.91	2.05	7.52	24.19	1.55	0.350
**Stigmasterol (mg/L)**	2.42	6.28	0.41	1.79	5.32	0.33	0.085
**Sitosterol (mg/L)**	7.46	23.37	1.38	5.89	30.63	1.47	0.137
**Lanosterol (mg/L)**	0.11	1.52	0.04	0.12	1.27	0.02	0.841
**Cholesterol (mg/L)**	1532.52	2765.64	357.84	1339.18	3197.45	245.73	0.825
**Desmosterol+Zymosterol+7-Dehydrocholesterol (mg/L)**	4.37	8.71	1.25	3.82	6.7	0.53	0.224

### PCSK9 and mortality

**Fig 1:**
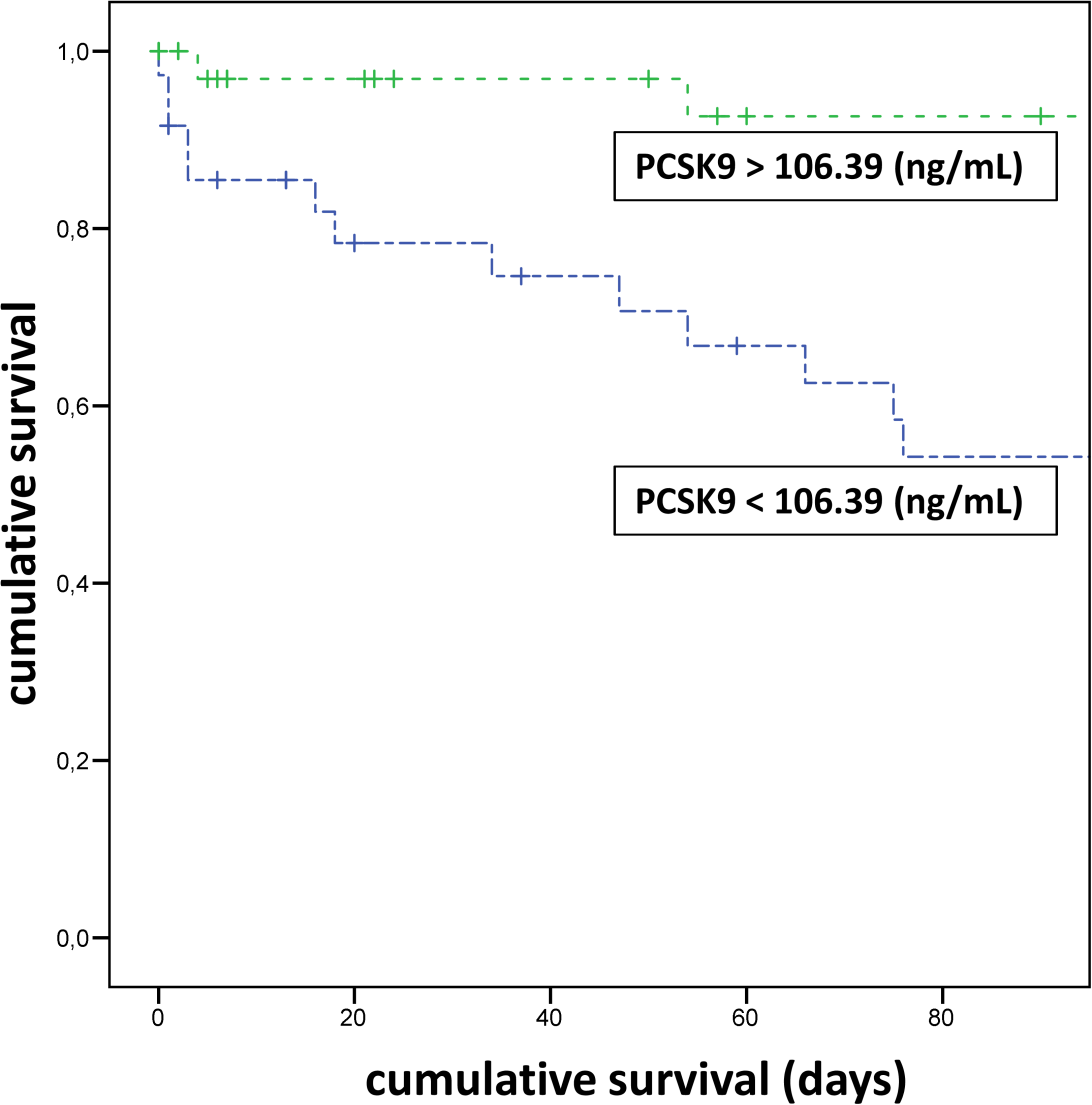
PCSK9 levels below the median (<106.39 ng/mL) were significantly associated with higher mortality during the 90-d follow-up (*p* = 0.002). (+ indicates censored patients due to liver transplantation).

Over a 90-d follow-up, 17 of the 74 patients underwent a liver transplantation. These patients were censored at the time of transplantation. Fifteen of the remaining 57 (26%) patients died within the 90-d follow-up (Supporting information: S1 Fig). Thirteen of 31 (42%) patients with PCSK9 levels below the median (<106.39 ng/ml) died compared to 2 of 26 (8%) patients with higher PCSK9 levels (*p* = 0.006). Fig 1 shows the Kaplan–Meier curves for PCSK9 > vs. < median. After the addition of the MELD score in the logistic regression analysis, PSCK9 levels were no longer significant (*p* = 0.817).

## Discussion

In the present study, low PCSK9 serum concentrations were associated with higher mortality in patients with end-stage liver disease. There was a strong negative correlation between PCSK9 levels and the MELD score. The underlying reason remains speculative, it could be suggested that lower levels of PCSK9 were due to decreased liver function and impaired synthesis of PCSK9. Impaired synthesis was supported by the correlations between PCSK9 levels and established parameters of liver synthesis, such as albumin, the INR, and cholinesterase. The negative correlation of PCSK9 and unconjugated bilirubin also hinted at impaired liver function.

The mean PCSK9 levels in the study population were much lower than those found in normal or healthy populations, with PCSK9 levels of healthy populations more than double the mean level of those with liver disease in the present study [24,25].

**Fig 2:**
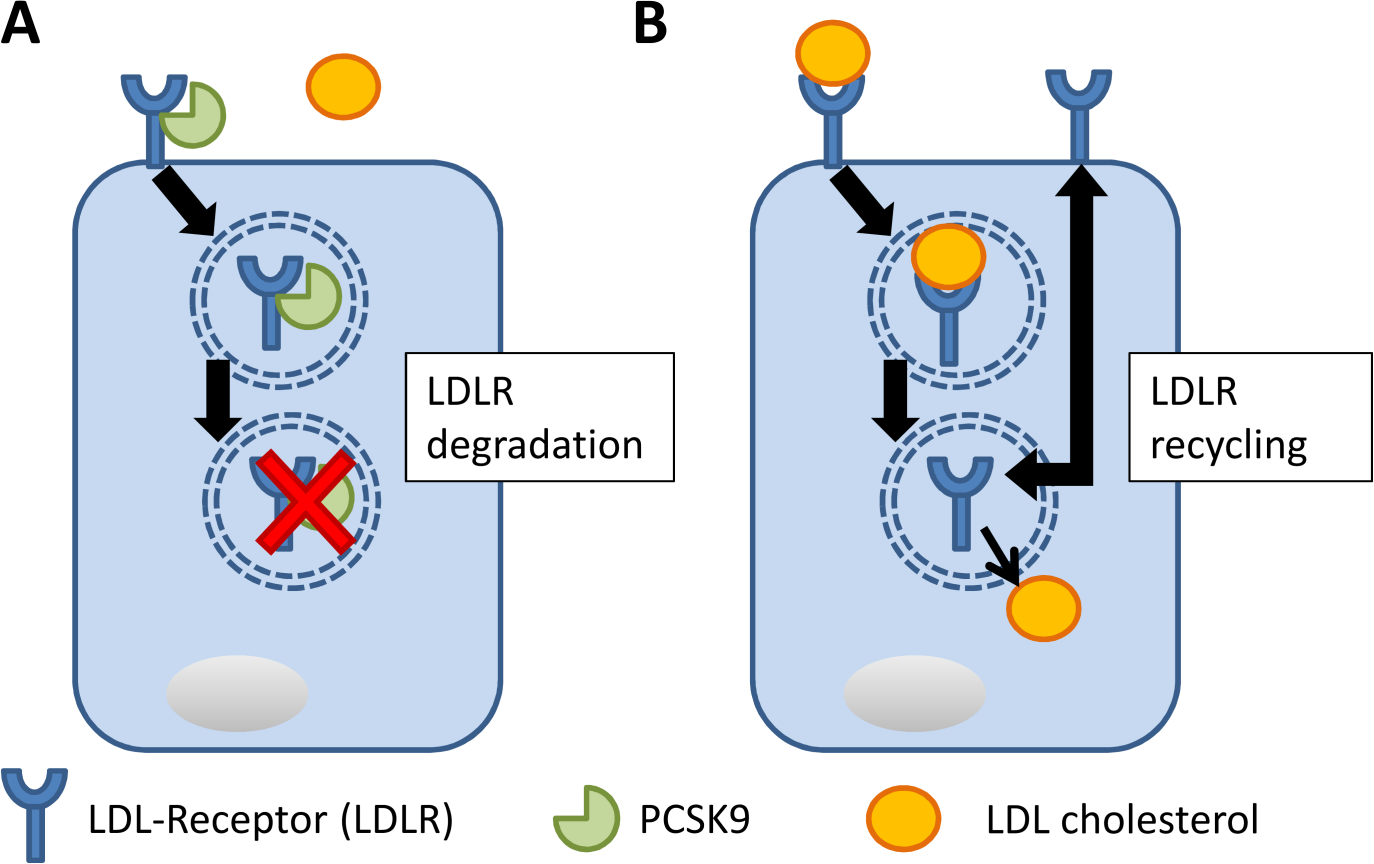
Scheme showing the interaction between PCSK9 and the LDLR on a hepatocyte. A: Available PCSK9 binds to the LDLR and results in internalization of the receptor, followed by lysosomal degradation. B: Without available PCSK9, LDL cholesterol is internalized by the LDLR into the hepatocyte. The LDLR circulates afterwards back to the surface.

Previous evidence suggested that low cholesterol levels in patients with liver cirrhosis were associated with a poor prognosis. Against this background, reduced PCSK9 concentrations could be a result of down regulation, as additional PCSK9 would further impair the cholesterol admission to the liver due to degradation of the hepatic LDLR (Fig 2). On the other hand, this could aggravate the lack of cholesterol for other tissues. Of note, a previous study showed that hepatic regeneration was significantly impaired in PCSK9- knockout mice and that the mice developed necrotic liver lesions, which were prevented by a high-cholesterol diet [3].

An alternative explanation for decreased levels of PCSK9 in association with advanced liver disease could be an accumulation of bile acids in the liver. This would lead to downregulation of both PCSK9 and LDLR by SREBP2 [9,10]. However, in the present study, conjugated bilirubin showed a weaker correlation with PCSK9 compared to the unconjugated bilirubin. Furthermore, there was no correlation between PCSK9 levels and alkaline phosphatase. In addition, with decreasing PCSK9 levels, those of γ-glutamyl transferase decreased significantly, providing no support for a link between PCSK9 levels in blood and cholestasis in these patients.

Given the absence of any significant correlations between PCSK9 levels and other sterols or their precursors, a (down-) regulatory effect of accumulated sterol derivatives on PCSK9 levels in this cohort appears unlikely. There were also no significant correlations between PCSK9 levels and phytosterols.

There was no significant association between the serum cholesterol concentration and PCSK9 concentration. This may indicate that other regulatory elements are responsible for the low cholesterol concentrations in patients with end-stage liver disease. The understanding of this regulation could be of relevance, as the underlying mechanisms for the association between higher mortality and lower cholesterol concentrations in these patients remain speculative [19,26].

This study has some limitations. Most importantly, the number of patients was limited, and the cohort consisted of liver disease of various etiologies. The study design was not suitable to clarify the pathophysiological effects of the serum concentration of PCSK9. In addition, some relevant parameters (i.e., LDLR content of hepatocytes, apo-lipoproteins, or bile acid composition) were not measured.

After the addition of the MELD score in the logistic regression analysis, PSCK9 levels were no longer significant for the prediction of 90-d mortality. However, the present study was designed to investigate the alteration in lipid metabolism in patients with end-stage liver disease and not to prove the predicting value of PCSK9 levels for mortality in addition to the MELD score. Further studies with more patients are needed to unravel a possible independent role of PCSK9 on mortality in end-stage-liver disease patients. However, our results clearly revealed that PCSK9 serum concentrations were associated to liver function and mortality.

In summary, the results of this study pointed to the disturbance of steroid regulation in patients with end-stage liver disease and an association of such disturbance with mortality. Further studies are required to acquire a more detailed understanding of the role of PCSK9 in liver-related mortality.

## Supporting information

S1 Fig Association between PCSK9 levels and MELD-Score (90-d follow-up). Green triangles: patients that received a liver transplant; blue rhombs: deceased patients that did not receive a liver transplant; red squares: surviving patients that did not receive a liver transplant.
(TIF)

S1 File PCSK9 patients raw data supplement. (CSV)
