# Supplementary figures and images for: Low PCSK9 levels are correlated with mortality in patients with end-stage liver disease
Source: PLoS One. 2017 Jul 20;12(7):e0181540. doi: 10.1371/journal.pone.0181540 (PMC5519179; doi:10.1371/journal.pone.0181540)

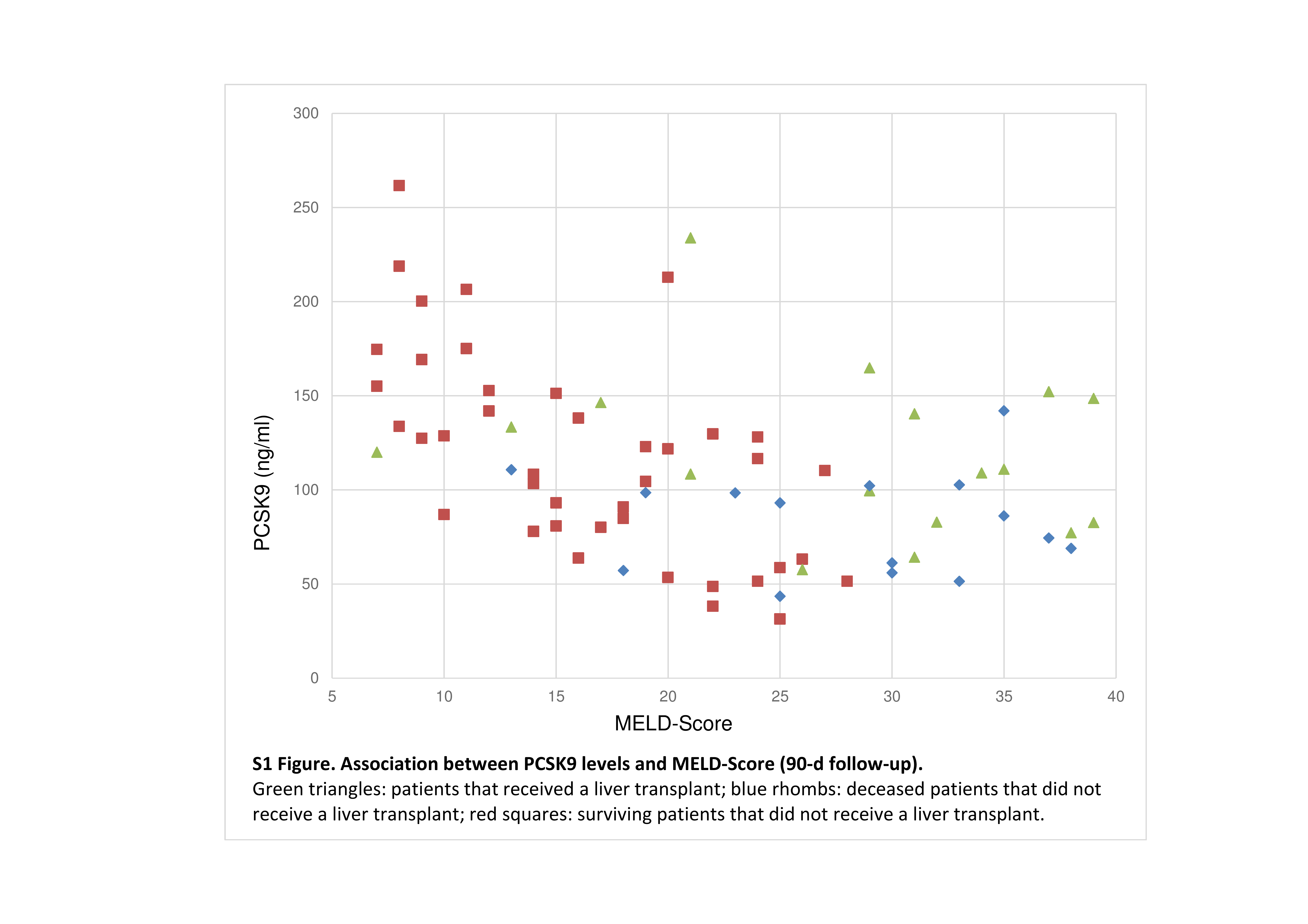

Supplement: S1 Fig — Green triangles: patients that received a liver transplant; blue rhombs: deceased patients that did not receive a liver transplant; red squares: surviving patients that did not receive a liver transplant. (TIF) [file pone.0181540.s001.tif]
